# Supplementary material for: Experiences with peer support for breastfeeding in Beirut, Lebanon: A qualitative study
Source: PLoS One. 2019 Oct 23;14(10):e0223687. doi: 10.1371/journal.pone.0223687 (PMC6808323; doi:10.1371/journal.pone.0223687)
Supplement: S1 Appendix — (DOCX) [file pone.0223687.s001.docx]

**Question Guide for In-depth Interviews**

**For breastfeeding mothers**

1. Tell me about your experience with breastfeeding with this birth.
2. How about your experience with receiving peer-support? What can you tell me about that?
3. What was the role of your support mother and how was the relationship with her?
4. How did the peer-support influence your relationships with other breastfeeding women in your social circle?

**For peer supporters**

1. Tell me about your experience with being a peer supporter.
2. In your opinion, what were your most important roles as a peer supporter?
3. What were the main challenges you experienced?
4. How did being a peer supporter influence your relationship with your own social circle?
